# Exploring the experiences of people with urogynaecology conditions in the UK: a reflexive thematic analysis and conceptual model

**DOI:** 10.1186/s12905-023-02592-w

**Published:** 2023-08-14

**Authors:** F Toye, S Dixon, M Izett-Kay, S Keating, A McNiven

**Affiliations:** 1 Physiotherapy Research Unit, Oxford University Hospitals NHS Foundation Trust, Oxford, OX37HE UK; 2 Nuffield Department of Primary Care Health Sciences, University of Oxford, Oxford, OX2 6GG UK; 3 Department of Urogynaecology, Oxford University Hospitals NHS Foundation Trust, Oxford, OX3 9DU UK

**Keywords:** Qualitative research, Urogynaecology, Women’s health

## Abstract

**Background:**

Urogynaecological conditions, such as pelvic organ prolapse, urinary incontinence, and urinary tract infection, can have a profound impact on people’s lives. The Independent Medicines and Medical Devices Safety Review highlights missed opportunities to prevent harm when patient voices are not incorporated into healthcare policy and practice. This resonates with the Women’s Health Strategy for England. The National Institute for Health and Care Research (NIHR) Policy Research Programme funded this in-depth qualitative exploration of people’s experiences of living with urogynaecological conditions, and of seeking healthcare treatment, to inform health and social care improvements in the UK.

**Methods:**

We conducted in-depth interviews online or by telephone (April 2021-December 2021) and used reflexive thematic analysis to develop themes that cut across urogynaecological conditions.

**Results:**

We spoke to seventy-four adults aged 22–84 across a range of backgrounds and lived experiences of urogynaecological conditions, including pelvic organ prolapse, urinary incontinence and persistent or recurring urinary tract infection. Eight themes were developed: [1] I get no respite from my own body; [2] I feel confined and separated; [3] I can no longer be ‘me’; [4] I am constrained by stigma, shame and silence; [5] I feel fragmented and lost in the healthcare system; [6] I need to be heard, believed, and valued; [7] I need respect as an equal partner in healthcare; and [8] (Re)connected to a more open community.

**Conclusions:**

High quality care focuses on the whole person rather than their body parts. Openness and candour support a shared decision-making model of care. A culture of shame can have a negative impact on access to health care and recovery.

## Background

Urogynaecological conditions, such as pelvic organ prolapse (POP) [1], urinary incontinence[2], and urinary tract infection (UTI)[3], can have a profound impact on people’s lives and identities. A review of qualitative research exploring people’s experience of POP highlights the negative impact of pelvic floor symptoms on personal identity, which is underpinned by a cycle of shame and silence [4]. A sense that pelvic floor symptoms are regarded as a “normal” part of having an (ageing) female reproductive body [4] resonates with qualitative findings showing that people with urinary incontinence find it difficult to decipher “am I ill or is this normal?” [5] For urinary incontinence, Toye and Barker highlight stigma, shame and guilt, feeding into a culture of secrecy that can become a barrier to healthcare [5]. Mendes and colleagues support the finding that bladder problems are regarded as a normal consequence of living in an (ageing) female reproductive body [6]. Similarly, qualitative research exploring the experience of urinary tract infection, indicate the profound effects on quality of life, along with an associated psychological toll, which is silently endured [7]. The profound impact of urogynaecological conditions further resonates with review findings in the context of core outcomes sets development for both urinary incontinence [8] and pelvic organ prolapse [9].

Public concern about the use of vaginal mesh to treat some of these conditions has highlighted unrecognised harm in healthcare from not hearing patients’ voices. Concerns due to adverse events such as pain, dyspareunia, incontinence, mesh exposure, haemorrhage, organ perforation, and infection[10–12] have led to governmental reviews, suspension of procedures, and litigation. In the UK, public concern was such that the use of vaginal mesh to treat urinary incontinence was suspended until further notice and thousands of women, and their health providers, face difficult decisions about surgical removal or expectant management. Following the Independent Medicines and Medical Devices Safety Review, the ‘First do no harm’ (or Cumberlege) Report highlighted missed opportunities for prevention of harm if women’s voices are ‘dismissed, overlooked and ignored’. The report emphasised the need to incorporate patient voices into healthcare policy and practice[13]: this resonated with the subsequent Women’s Health Strategy for England[14]. Following the publication of the Cumberlege report[13], the National Institute for Health and Care Research (NIHR) Policy Research Programme funded this in-depth qualitative exploration of people’s experiences of living with urogynaecological conditions, and of seeking healthcare treatment, to inform health and social care improvements in the UK. We report findings from this study and highlight important themes for service development.

## Methods

The study was designed and analysed with the input of ten patient and public involvement (PPI) advisors who had lived experience of urogynaecological conditions. This study took place in the UK, with interviews conducted between April 2021 and December 2021. We recruited UK adults living with urogynaecological conditions through multiple sources: NHS sites; advertisements, support groups, social media, and advisory panel members; snowball sampling; and working with health advocacy organisations and groups focused on inclusive gynaecological health care.

### Qualitative data collection

Three experienced social scientists conducted qualitative semi-structured interviews, either as online Microsoft Teams video calls or by telephone. They invited participants to tell them about their experiences and used a topic guide to prompt discussion. The guide was developed from qualitative research syntheses[4, 5, 7, 15] in collaboration with an advisory group, and was piloted on two people with lived experience. The interviews started with an invitation for participants to tell their story in their own way – “I’m going to ask for you to tell me a bit about your experiences with (…). You can say as much or as little as you want, and you can start at any point in your story”. With consent, all interviews were recorded and transcribed verbatim by approved professional transcribers compliant with data security policies. The researchers checked each transcript against the recording for accuracy, and removed names and places for de-identification. Participants had the opportunity to read and mark any sections which they would like to expand or redact, and were sent a summary of the key aspects they shared with us in their interview. This form of respondent validation[16] is a strategy used to ensure trustworthiness of research.

### Analysis

The research team used the six stages of reflexive thematic analysis to develop themes that cut across participants: (a) familiarisation; (b) coding; (c) generating initial themes; (d) developing and reviewing themes; (e) refining and naming themes; (f) writing up. This provides a flexible method for distilling data into themes organised around a central idea[17]. Reflexive thematic analysis goes beyond topic description and values the researchers’ interpretive lens. Interview transcripts were uploaded to NVivo 12 Software for qualitative analysis. Two authors coded each transcript, a process that involves assigning a short phrase to a unit of meaning. These two researchers then discussed whether the assigned code did justice to the meaning. The aim of discussion was not to agree on a ‘correct’ code but to make sure that valuable nuance was not lost. The codes were then organised into themes around a central idea. Working in close collaboration during weekly meetings, the research team developed themes through constant comparison and discussion. This process facilitates the development of ideas by comparing similarities and differences across data[17]. We worked with ten PPI advisors to refine the themes. Our analysis was influenced by conceptual approaches to qualitative research[18], which aim to offer insight through theorising. Again, working as a team during a series of meetings, we designed a conceptual model that integrated the themes into a storyline.

## Results

**Fig. 1:**
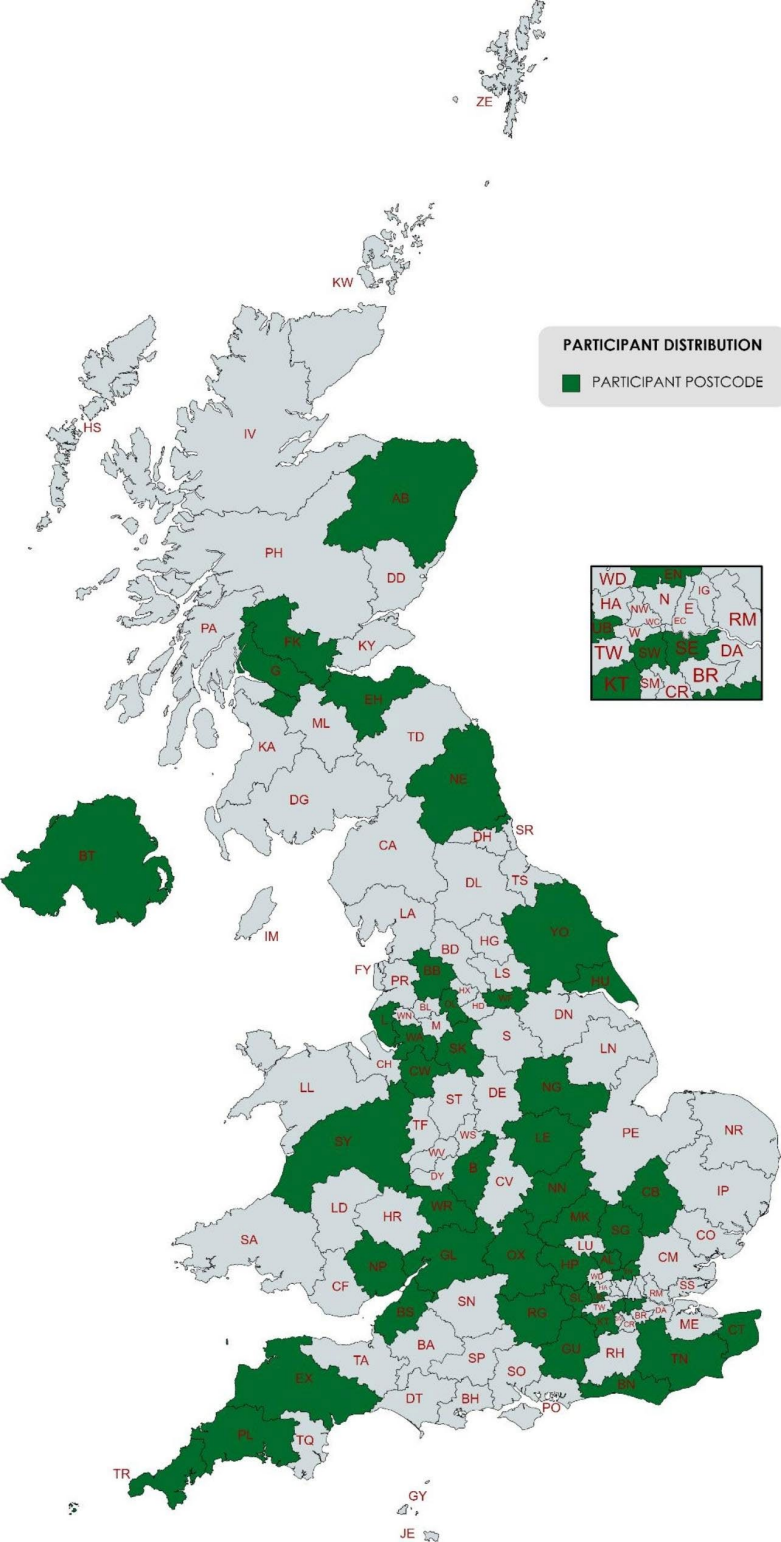
Geographical distribution of participants from postcode data

**Fig. 2:**
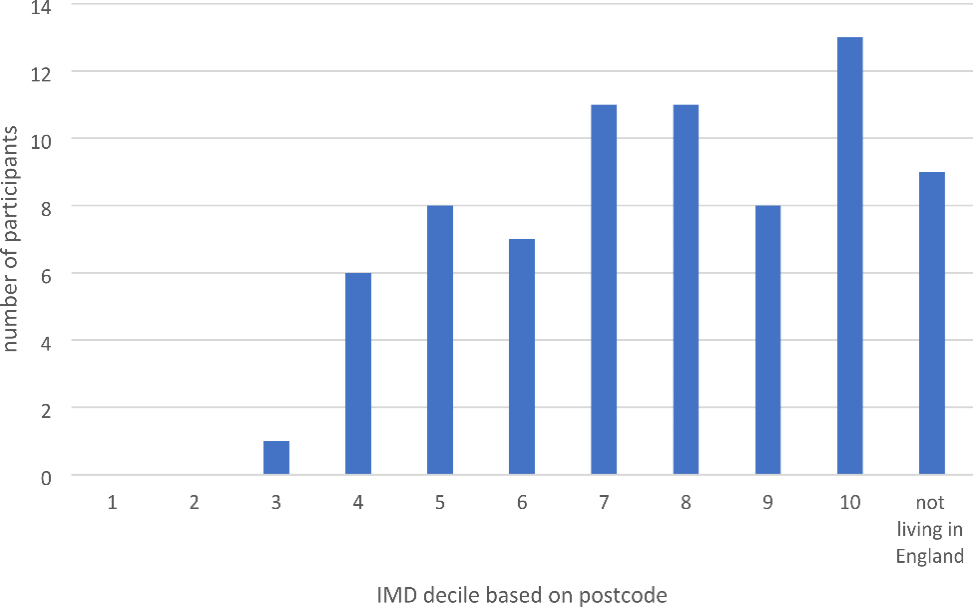
Number of participants in each decile of deprivation as indicated by the Index of Multiple Deprivation (IMD) for postcode

**Fig. 3:**
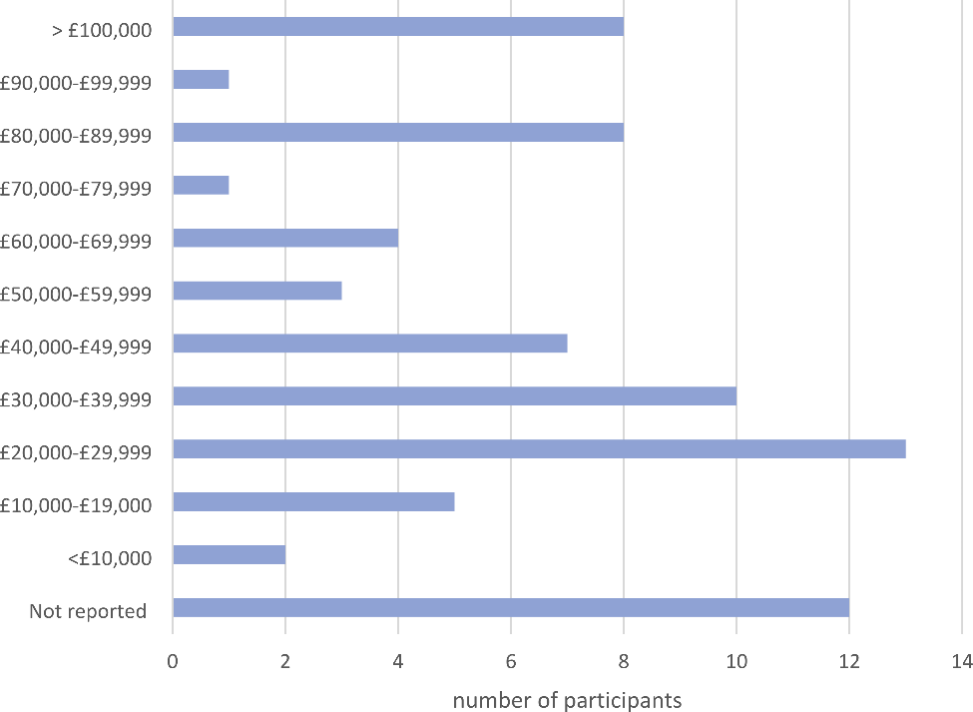
Annual household income (self-reported)

**Fig. 4:**
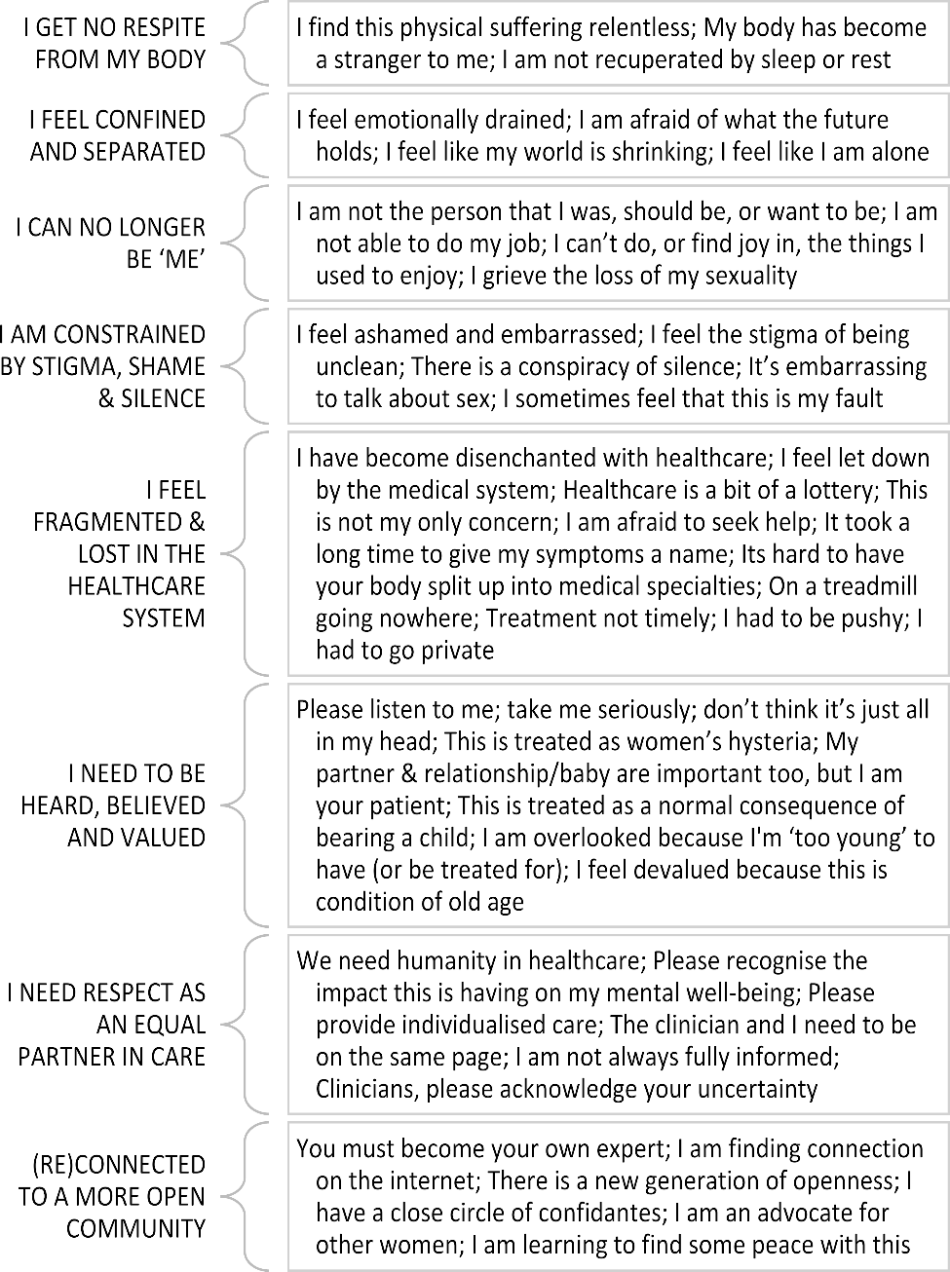
Organisation of subthemes into final eight themes

**Fig. 5:**
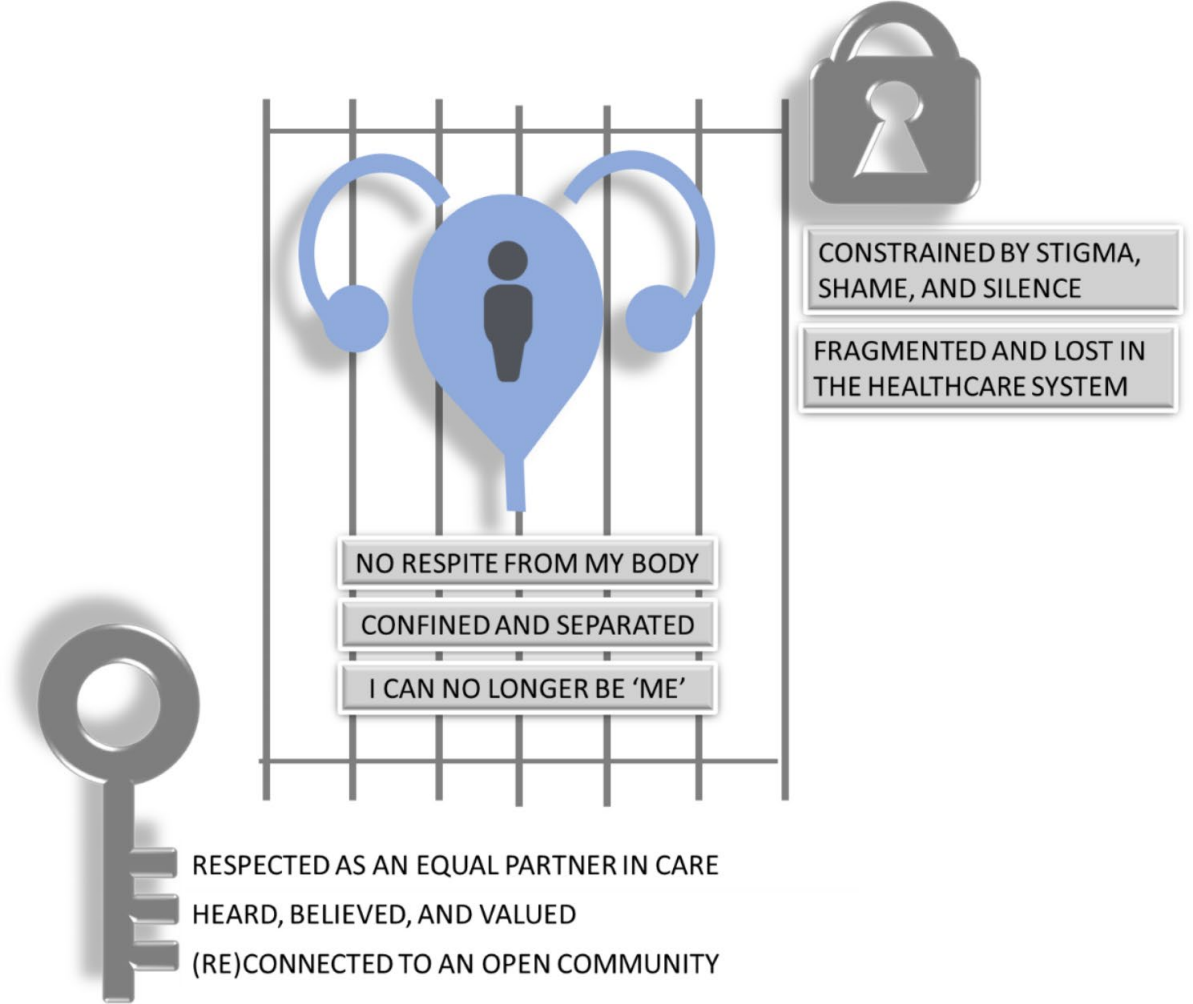
Conceptual model constructed collaboratively from eight themes

Seventy-four people gave written consent to use their interview data in analyses. Interviews lasted between 44 and 138 min. Descriptive characteristics of the participants are shown in Table 1. Our sample were from a range of UK locations, including ten participants from Wales, Scotland, and Northern Ireland (Fig. 1). The Index of Multiple Deprivation (IMD), based on the postcode, ranged from 7309 to 32,758 (mean 22,384). IMD ranks neighbourhoods in England from 1 (most deprived area) to 32,844 (least deprived area) based on several indices of deprivation. Fifteen participants were located within the 50% most deprived areas of England, and fifty lived within the 50% least deprived areas (Fig. 2). No IMD data is available for participants living outside England. Twenty participants were living on an annual household income of less than £30,000 (Fig. 3). Fifty-three participants had been awarded a university degree. The conditions represented include urinary incontinence (n = 36), urinary tract infection (n = 25), pelvic organ prolapse (n = 35), with twenty-nine participants experiencing two or more of these conditions, and a subcategory who had undergone vaginal mesh surgery (n = 18). Figure 4 shows how we refined the data into 48 initial ideas, and then into 8 final themes. Figure 5 illustrates our conceptual summary of themes.

**Table 1:** Descriptive characteristics of participants

		Count	% (mean)
**Age (years)**		22–84	(52)
**Country of residence**	England	64	86%
	Northern Ireland	1	2%
	Scotland	6	8%
	Wales	3	4%
**Gender identity**	Female	73	99%
	Non-binary	1	1%
**Ethnicity**	White - British	58	78%
	White - Other	11	15%
	Arab	2	3%
	Latin	2	3%
	Asian	1	1%
	Black	0	0%
**Relationship status**	Single	5	7%
	Partnered but not cohabitating	4	5%
	Partnered and cohabitating	59	80%
	Divorced	4	5%
	Widowed	2	3%
**Has children**	Yes	61	82%
**Highest level of education**	GCSE	5	7%
	A-level/vocational training	14	19%
	Bachelor’s degree	26	35%
	Advanced degree	28	38%
	Did not provide	1	1%
**Employment status**	Full-time employed	16	22%
	Part-time employed	5	7%
	Student	4	5%
	Long-term leave	3	4%
	Not in paid employment (1 carer)	12	16%
	Retired	22	30%
	Self employed	7	9%
	Maternity leave	5	7%
**Type of accommodation**	Privately owned	59	80%
	Rented	7	9%
	Shared (one with family)	2	3%
	Other (2 council homes)	4	5%
	Not reported	2	3%
**Condition(s)**	Urinary Incontinence	36	% not given as some had two or more conditions
	Urinary Tract Infection	25	
	Pelvic Organ Prolapse	35	
**Disability**	Yes	35	47%
	Not reported	1	1%

### I get no respite from my body

Across urogynaecological conditions, participants described “relentless”, meaning persistent and punishing physical symptoms: “it is excruciating”; “it is like barbed wire”; “it is agony”; “it drags”; “it pulls”. Symptoms affected quality of sleep, rest, and the ability to recuperate. There was a sense of being physically exhausted by a “broken” body.

Just absolutely constant […] I couldn’t do anything with my kids. I couldn’t enjoy anything. […] It’s absolutely awful. I mean, there was no life at all […] Even once the pain comes off, it’s the kind of constant awareness like you can’t just have a normal day. (P27, UTI)

For some, a body that had been silent had become ever present: the body did not feel the same or had become an unwelcome stranger. There was a sense of being let down by a body that was no longer reliably comfortable to inhabit.

[It felt] like a huge kind of empty cavernous space where things can fall out of and you can’t close [it] and a kind of pulling and a tiredness. […] A discomfort that is with every step, every second, like you can’t forget about it. You’re just thinking about it constantly. (P19, POP)

There were participants who were experiencing an accumulation of health and life concerns which contributed to the experience of a painful and ever-present body. Physical suffering or discomfort was further compounded by social expectations to be able-bodied, as one participant described:

There’s definitely a lot of [pressure from] culture. […] People think ‘I haven’t seen a GP for 30 years’ […] [and that attitude] really has an effect on people who that’s just not their reality where they can go around as if they don’t have a body. (P35, UTI)

### I feel confined and separated

Participants described symptoms as being like a “leash” that constrained their geographical and social space, and some feared the impact of this on their future. Managing vaginal, bladder or bowel symptoms became the central pivot around which participants had to carefully plan their lives, meaning spontaneity was lost.

I just want to be at home, be in my pyjamas and be comfortable. […] I don’t want to have to dress up. […] I don’t like to have to put a smile on my face when I’m not smiling. […] It’s just this state of limbo […] I can’t seem to move forward at all. (P45, UI, mesh surgery)

Some felt alone because it was so difficult to tell other people about what they were going through. Participants retreated into an internal space, sometimes consumed by their losses. Thoughts and feelings could lurk in the background, like “a boil building up in my head”.

When you’re exhausted, that drags your emotions down. […] You just want to shut the whole world out and not have anybody come round or anything, but you have to because you’ve gotta live. I do have to force myself to do stuff sometimes. (P48, UI, POP, mesh surgery)

Participants feared what the future might hold and the impact of future pregnancies, menopause, and ageing. Their world had become unpredictable, and there was a sense of being emotionally drained by uncertainty and worry.

## I can no longer be ‘me’

There was a sense of no longer being the person that you once were, should be, or wanted to be. Participants told us that they could no longer do, or find joy in, things that they once enjoyed. Some found it challenging to maintain valued social roles and struggled with feeling more reliant on other people. Some found it difficult to keep working and worried that colleagues might think that they were “lazy”. These losses were exacerbated if work was integral to personal identity. Participants described feeling less than they once were.

You want to be the fun person that you were and now you’re wrapped up in this miserable cloak that sort of shrouds you. […] I used to go out to with a group of friends. […] I was really active. […] [Now] I sort of hibernate in the house. […] I’m just a completely different person. (P53, UI, mesh surgery)

For some, their sense of self was affected by the impact on intimate relationships. Some avoided or dreaded sex, or felt that they were not “like a woman anymore”.

It affects your relationships, all of that is going to eventually have a knock-on effect on you emotionally. I feel like I’m not the person I used to be. […] I don’t feel attractive anymore. I feel like I’ve lost myself quite a lot. (P8, POP, mesh surgery)

### I am constrained by stigma, shame, and silence

Participants felt stigmatised and ashamed for several reasons: for leaking, for having a “broken” body, for feeling “unclean”, or for simply “being old”. The feeling of shame and taboo was exacerbated by advice from others about cleanliness.

Anything affecting your genital tract is kind of a bit of a taboo topic. […] I’d been made to feel ashamed and responsible. […] Someone examining me and saying it was “disgusting” […] I kind of can’t really believe that a […] consultant would talk to any [patient] like that. (P17, POP)

Participants felt mortified about body leakage, and this perpetuated silence. The link between their symptoms and their sexual organs, sexuality, sexual encounters, and intimacy compounded feelings of shame. Some were left feeling like people thought it was their fault, and some even blamed themselves.

It feels a bit shameful. […] I don’t want to talk about it with that many people because it’s, you know, it’s my vagina and it’s my sexual function, you know, bladder function, it’s kind of all of these parts of ourselves and affect issues that we tend not to talk about. (P54, POP)

Participants recognised that silence could remain a barrier to knowledge and effective care. There was also a sense that stigma would prevent people from seeking care. Some felt that you had the right to know things that others (mothers, sisters, teachers, health providers, the media) had kept from you, and some felt a strong sense of injustice and a duty to break the “conspiracy of silence”.

### I feel fragmented and lost in the healthcare system

Participants felt fragmented by a health system that hinged on naming and diagnosing. If symptoms remained un-named (or were contested), there was a feeling that you were not legitimately ill. The diagnostic process itself could be frightening, long-lasting, and yet lead nowhere.

I had every kind of test you could imagine: really invasive, unpleasant tests. […] You fill your bladder with water, and you’ve got to hop up and down while somebody taps your nether regions to see if you’re leaking, [ergh] very pleasant. (P42, UI, POP, mesh surgery)

There was a sense that, as patients, people became fragmented into medical specialties (gynaecology, urogynaecology, urology, colorectal, midwifery, pain services) whereas, in their lived experience, boundaries blurred and conditions did not always map neatly onto body parts or body systems. This fragmentation could be experienced as “depersonalising” and “dehumanising”.

[Medics have a] disengaged notion of the human body and de-personalise it, but I think in a way that’s the problem. This is so personal, so intimate, and so connected to our cultural ideas of dirt and shame, and cleanliness, womanhood, and sexuality […] that it’s almost like the opposite, you don’t want to be de-personalised at all. (P26, UTI)

Healthcare was described as a lottery where information could be conflicting, and where services “don’t talk to each other”. Some therefore felt forced to become “pushy” or to seek a private consultation. There was a sense of being “passed from pillar to post”.

Having to go through your whole history each time, it’s awful, and one doctor will say one thing, another doctor will say another. So, you’re kind of like left in the middle. Well, who do I listen to? (P36, UI, mesh surgery)

Whilst recognising that there were many health providers who did care, and who were doing their best in the most challenging circumstances, some felt badly let down and had lost trust. Some had disengaged from healthcare system for a range of reasons: they were unable to sustain the time and energy burden; they did not want to risk being let down again or being disregarded; they were concerned that they might receive a life-changing or life-threatening diagnosis, painful examinations, or unwanted treatments; they felt worn down from navigating healthcare.

### I need to be heard, believed, and valued

Participants described feeling “fobbed off”, undermined, trivialised, or not believed. There was a sense that urogynaecological conditions were treated as a *natural*, and therefore normal, function of pregnancy, childbirth, menopause, and ageing. Whilst recognising that clinicians might normalise symptoms to reassure patients, this was experienced as a form of trivialisation. Some felt ignored or disregarded following traumatic vaginal births, or felt as if they had been treated like “just another old woman” with a bladder, bowel, or pelvic condition. Some felt a strong sense of injustice or inequity that underpinned being female.

It wasn’t something that I did to myself, and this attitude of ‘you chose to have children’ isn’t fair and I can’t help thinking that had it been […] a footballing injury that a man had, […] there would be acknowledgment of his pain […] [and he would be] respectfully treated. (P70, POP)

Some felt that health providers “wedged your symptoms” into a diagnosis or treatment and that, if you did not fit, you might be sent on your “merry way” to cope alone. Some left consultations feeling as if they were making a fuss, or that they were “neurotic” or “hysterical”. Some used the term “gaslighting” to describe perceived attempts to make them “question their own reality”.

From a doctor’s point of view, I think they may be thinking ‘oh well, here we go again, some hypochondriac patient, she’s got this, that and the other, she’s got everything under the sun in the medical book’. (P39, UI, POP, mesh surgery)

Some felt devalued because their needs had been side-lined for that of their child (or partner), and wanted their own needs to be taken more seriously: “I am your patient”.

[I felt] that all the system cared about was the baby. And actually, the baby was out, happy and safe and healthy, and I was left injured and broken and not well, and nobody really minded, and nobody would provide the support that I required. (P19, POP)

### I need respect as an equal partner in healthcare

Participants felt that their humanity and broader lives should feature in their healthcare interactions and decisions: *caring* should be integral to health*care*. Participants wanted clinicians to recognise the impact of symptoms on their emotional well-being whilst also validating their experience as “real” and physical. One participant told us how important it was to feel that the health provider was alongside you as a fellow human-being.

It’s about humanisation. […] Recognise that this is an intimate, dark, shaming space and that’s not our fault, it’s society’s fault, culture’s fault, and therefore cross that boundary, come in with us, be in that space where, where women want to be talking about difficult stuff. (P26, UTI)

There was a sense that a good healthcare provider incorporated a person’s unique circumstances and views, rather than being “paternalistic” or “controlling”.

I felt quite vulnerable because they would be using words that I didn’t understand, but I didn’t have the confidence to say, “What’s that?” or, “Stop, I don’t understand” or, “Why is this happening?” or, “What alternatives?” (P52, UI, POP, mesh surgery)

Good care also meant fully informing patients about their condition and all possible treatments, acknowledging medical uncertainty, being open to new ideas and realising that best practice recommendations inevitably change over time. Whilst recognising the daunting amount of information and the desire to not “scare” patients, there was a strong sense that being “misled” (or even “lied to”) by health providers was harmful.

### (Re)connected to a more open community

Participants felt an obligation to do their own research because information and support had been inadequate, or because they wanted to (re)gain a sense of control. Participants found knowledge and support online, which helped them feel less alone. Some recognised that they were now operating in a new generation of openness concerning women’s health, a place where conventional wisdom was facing challenges.

What I found on the internet was a complete change in the narrative around what you could do if you had prolapse, and challenging just the conventional wisdom I think about what it means to have a prolapse. […] [I] would not be here today without that valuable support. (P56, POP)

There was a sense of camaraderie and peer-advocacy. Participants worried about family, friends, and others who they felt were worse off, or were less confident to speak out. Older participants, realising sometimes for the first time that “young women get this”, worried about them; just as younger participants worried about older women who they felt might become isolated or dependent on others, recognising that older women might be from “a generation that did not talk about it”.

### Conceptual summary

Figure 5 illustrates our conceptual summary of findings. At the centre of our model, we illustrate a place of constraint where [1] I get no respite from my body, where [2] I feel confined and separated from others, and where [3] I can no longer be ‘me’. This constraint is maintained, not only by [4] stigma, shame, and silence, but also from feeling [5] fragmented and lost in the healthcare system. We illustrate that being [6] respected as an equal partner in care, [7] heard, believed, and valued, and finding a way to [8] connect or reconnect to an open community of people who share my experiences, are important means to unlocking the door to better healthcare experiences.

## Discussion

Our findings highlight the profound effects of urogynaecological conditions on a person’s sense of self and well-being, and that experiences of healthcare can be far from ideal. We highlight sociocultural experiences that impact on care: first, a cycle of stigma, shame and silence that can lead to isolation and prevent care-seeking: second, patients found that they needed a medical diagnosis to access care and to validate experience, and that this process can be difficult to navigate because of fragmentation of care into specialties.

We used established methods of qualitative analysis to develop themes from 74 interviews with people with a range of lived experience from a wide geographic and socio-economic context. The strength of qualitative research is to draw on purposive samples to give voice to lived experience, and to develop ideas from information rich samples[17]. Limitations were that most participants were white cisgender women, living in privately owned accommodation with a partner and at least one child. We interviewed participants during the COVID-19 pandemic when access to services was curtailed. Although a relatively large proportion of participants had undergone mesh surgery, the themes that we report cut across conditions and may also be transferable beyond this sample.

The research team brought different perspectives in terms of age (20’s to 50’s), profession, social science, and clinical disciplines, and lived experience. Personal perspective is integral to reflexive thematic analysis and this is not a limitation for interpretive paradigms[17]. Nonetheless, good qualitative research should interrogate subjectivity. Our team philosophy was non-hierarchical, and our PPI members served as critical friends to ensure rigour and relevance.

### Interpretation

Our findings resonate with qualitative research highlighting identity loss, reduced participation, and shame[4, 5, 7, 19–21]. They describe a biomedical system where people experience healthcare as a *patient with a body*, rather than an *embodied person*: this indicates a need for holistic care. There is an important distinction between *disease* (named pathological entities) and *illness* (what the patient feels) [22]. Qualitative research reminds us that it is an embodied person with an ‘illness’ that seeks help. Our findings resonate with studies highlighting the burden of suffering with ‘un-named’ conditions where validation and treatment rely on diagnosis[23, 24].

Our findings also highlight specific aspects of provider-patient interactions that would support re-structuring services to meet experiential needs rather than purely medical ones. For example, the importance placed upon when my clinician does (or does not): respect me as a person; listen and understand me; take me seriously; consider my preferences and priorities; give me the information that I need; take time to be open and honest. This suggests incongruity between the patient perspective and the systems and services designed to address their needs. Our findings re-iterate the needs for patients to be informed. The UK General Medical Council (GMC) affirms that patients have the right to be involved, and supported, in treatment decisions, and encourage doctors to discuss and find out what matters to patients, rather than make assumptions about what needs to be known[25]. In the context of treatment morbidity from mesh surgery[13], the failure to recognise, acknowledge and apologise for harm is an important message, supported by our data. The clinician’s duty of candour stipulates the need to be open and honest if things go wrong. Guidance from the GMC and Nursing and Midwifery Council confirm that apologising is not admitting legal liability, and that ‘saying sorry is the right thing to do’[26]. It may be that fear of reprisal has been a strong barrier to candour that has contributed to ‘harm’. From a clinical perspective, our findings indicate the potential harms from interventions. The experiences of women from this sample who attributed complications to vaginal mesh surgery raises some important issues for healthcare education, policy, and practice in the context of surgical decision-making [27]. Ongoing developments in the field of urogynaecology include an array of non-surgical approaches such as laser therapy[28], as well as evolving surgical techniques to avoid mesh prosthesis[29] such as periurethral bulking for stress incontinence[30] and native tissue repair for prolapse[31]. Whilst not explicit, this may reflect increasing partnership with patients and valuing their voices in development of novel therapies. New surgical strategies should be thoroughly evaluated to determine impact on quality of life [32].

A recent review[33] recommends the inclusion of patient perspectives in the assessment of pelvic-floor symptoms and the NIHR has recently commissioned a study to develop a PROM for use in evaluating the outcomes of surgical treatment of pelvic organ prolapse and stress urinary incontinence (NIHR - HTA 21/583). Our findings highlight issues that would be important to consider. Mishler reminds us that the purpose of asking questions is, quite simply, *‘to understand what respondents mean by what they say’*[34]. As such, it is essential to remember that quantifiable outcome measures can only ever be a proxy for lived experience.

## Conclusions

Our findings highlight the impact of shame and stigma on health and recovery. Shame is embedded in socio-cultural experiences that transcend outcome measurement and require wider social change. Our findings support the need for education and openness about urogynaecological conditions and indicate that a culture of shame can be detrimental to treatment access and outcome, and has the potential to cause harm. Recent media coverage indicates a positive move towards openness. Further research is needed to explore the nuanced experience of marginalised groups.

## Data Availability

Data for this project are not currently available for access outside the study research team. The dataset may be shared when finalised to bona fide researchers following request to the AM, the principal funding applicant.

## List of abbreviations

NIHR: National Institute for Health and Care Research (NIHR)
IMD: Index of Multiple Deprivation (IMD)
UTI: Urinary Tract Infection
POP: Pelvic Organ Prolapse
UI: Urinary Incontinence
GMC: General Medical Council
PROM: Patient Reported Outcome Measure
